# Predictors of knowledge of H1N1 infection and transmission in the U.S. population

**DOI:** 10.1186/1471-2458-12-328

**Published:** 2012-05-03

**Authors:** Elena Savoia, Marcia A Testa, Kasisomayajula Viswanath

**Affiliations:** 1 Department of Biostatistics and Division of Policy Translation and Leadership Development, Harvard School of Public Health, 677 Huntington Avenue, Boston, MA 02115, U.S.A; 2 Department of Society, Health and Human Development, Harvard School of Public Health, and Dana Farber Cancer Institute, 677 Huntington Avenue, Boston, MA 02115, U.S.A

**Keywords:** H1N1, Communication, Socioeconomic position, Neighborhood cohesion, U.S.A., Race, Knowledge gaps, Survey

## Abstract

**Background:**

The strength of a society’s response to a public health emergency depends partly on meeting the needs of all segments of the population, especially those who are most vulnerable and subject to greatest adversity. Since the early stages of the H1N1 pandemic, public communication of H1N1 information has been recognized as a challenging issue. Public communication is considered a critical public health task to mitigating adverse population health outcomes before, during, and after public health emergencies. To investigate knowledge and knowledge gaps in the general population regarding the H1N1 pandemic, and to identify the social determinants associated with those gaps, we conducted a survey in March 2010 using a representative random sample of U.S. households.

**Methods:**

Data were gathered from 1,569 respondents (66.3% response rate) and analyzed using ordered logistic regression to study the impact of socioeconomic factors and demographic characteristics on the individual’s knowledge concerning H1N1 infection and transmission.

**Results:**

Results suggest that level of education and home ownership, reliable indicators of socioeconomic position (SEP), were associated with knowledge of H1N1. Level of education was found to be directly associated with level of knowledge about virus transmission [OR = 1.35, 95% C.I. 1.12-1.63]. Home ownership versus renting was also positively associated with knowledge on the signs and symptoms of H1N1 infection in particular [OR = 2.89, 95% C.I. 1.26-6.66].

**Conclusions:**

Policymakers and public health practitioners should take specific SEP factors into consideration when implementing educational and preventive interventions promoting the health and preparedness of the population, and when designing communication campaigns during a public health emergency.

## Background

The strength of a society’s response to a public health emergency depends partly on meeting the needs of all segments of the population, especially those who are most vulnerable and subject to a disproportionate share of adversity. Failing to address the great diversity of special health and medical concerns, language and cultural barriers, and other life circumstances could decrease the effectiveness of the public health response to specific threats, including reducing the benefits of timely interventions. Although the adverse impact of the H1N1 pandemic was not as severe as initially expected, it provided an opportunity to test community preparedness, particularly the ability of communities to prepare for, withstand, and recover from public health incidents in both the short and long term [1].

From the earliest days of the H1N1 outbreak in spring 2009, the U.S. government acknowledged that public communication would be challenged by the uncertainty of the unfolding circumstances of a potential pandemic [2]. Familiar messages that mirrored advice for reducing contagion from common colds and flu were given, including basic hygiene measures (e.g., hand-washing, coughing etiquette, etc.) and ways to contain the spread of infection (e.g., staying at home with flu-like symptoms, deferring non-essential travel, and keeping children at home during school closures, etc.). However, policy recommendations and communication messages had to be developed in the absence of accurate data on the magnitude and severity of the outbreak. Consequently, information was often communicated under conditions of uncertainty, and with very limited knowledge on how people would react and respond to public messaging. Complicating this situation was the reality that this information was directed at social groups known to vary widely in their capacity to follow specific public health advice because of broad disparities in underlying health status, socioeconomic status, communication abilities, and health literacy [3,4]. The result was communication inequalities, which are defined as differences in social groups in accessing and using health information and the consequential effects of such differences, including knowledge and behaviors [4]. It has been widely documented that social determinants such as social class, neighborhood conditions, social capital, and race/ethnicity are strongly associated with health outcomes and inequalities in communication, including knowledge gaps [5-7].

During the H1N1 pandemic, federal, state, and local public health agencies across the U.S. engaged in a variety of public communication efforts to inform the population, encourage the adoption of preventive behaviors, and limit the spread of the disease. A poll conducted by the United States’ Centers for Disease Control and Prevention (CDC) in late April 2010 showed that, overall, most of the U.S. public had a fairly clear understanding of what H1N1 flu was and how to prevent infection [8]. However, to date, little research has focused on the differences in knowledge and communication inequalities experienced by various social groups during this event. The aim of this study was to investigate gaps in knowledge experienced by the U.S. population during the 2009–2010 H1N1 pandemic and to identify the social determinants associated with such gaps. Specifically, we tested the hypothesis that respondents with lower socioeconomic position (SEP) were more likely to have less knowledge about H1N1 in terms of virus transmission and signs and symptoms of infection than those with a higher SEP. Our hope was that findings from this study could help inform policymakers and practitioners developing communication campaigns during public health emergencies.

## Methods

### Description of the survey instrument

During December 2009 through January 2010, we developed a survey instrument to investigate information sources, knowledge, and attitudes about H1N1 in the U.S. population. Prior to developing the survey, we conducted a series of focus groups in Massachusetts to assess people’s sources of information, credibility attached to the sources, barriers to obtaining and processing the information, and H1N1-related knowledge and behaviors. To develop the survey questions, we used the “*structural influence model of communication in public health emergency preparedness (PHEP)”* to guide underlying theoretical assumptions [9]. We then finalized the questionnaire after cognitive debriefing and a pilot test with twenty-five individuals, and fielded it between March 16^th^ 29^th^ 2010 among a representative random sample of U.S. households.

### Description of the sample

A representative sample of U.S. households was selected by the survey research firm, Knowledge Networks using their KnowledgePanel® online survey methodology. The selected panel of survey respondents was based upon a representative sample of U.S. adults using a dual sampling frame: a random digit dial sample as well as an address-based sample, a strategy that includes both landline and cell-phone-only households [10]. Knowledge Networks provides selected households with access to the Internet and hardware if needed. Post-stratification weights were used to adjust for non-coverage and non-responder biases by applying the most recent data from the Current Population Survey and the 2006 Pew Hispanic Center of Latinos [11].

Post-stratification weighting included gender, age, race/ethnicity, education, census region, urbanicity, Internet access, and dominant language. The survey was conducted in both English and Spanish. For the purpose of this study, we only analyzed questions focusing on knowledge about H1N1. We obtained institutional review board (IRB) approval to conduct this study from the Human Subjects IRB at the Harvard School of Public Health.

### Measures

#### Dependent variables

We devised a scoring system to yield two dependent variables:1) knowledge about H1N1 transmission and 2) knowledge about signs and symptoms of H1N1 infection.

To determine the first of these, we asked: *1) To the best of your knowledge how can someone get H1N1?* Response options were:* from being in close contact with someone who has H1N1 (within arm’s length of someone), from eating pork, from coming in contact with pigs, from touching objects recently touched by someone with flu, none of above. For* the second dependent variable, knowledge on the signs and symptoms of H1N1 infection, we asked: *To the best of your knowledge, what are some of the most common/ likely symptoms of H1N1?* Response options were*: coughing, fever, body aches, bleeding, rash, stomach pain,* and *chest pain.*

Given that each question had multiple-choice responses, including both right and wrong response options, we used patterns of subject responses rather than individual responses to build the dependent variables. For the first question respondents could obtain a score of 0, 1, or 2. A score of 2 was given if the following correct answers were checked: “*close contact”* and “*touching objects*,*”* and none of the following wrong options were checked: *eating pork* and *none of above.* A score of 1 was given if either one of the two correct options and none of the wrong ones were checked. A score of 0 was given to any other combination of responses. For the second question, respondents could obtain a score of 0, 1 or 2 as well. A score of 2 was given if the following three correct answers were checked: *coughing, fever,* and *body aches* and none of the following wrong options were checked: *rash* and *bleeding.* A score of 1 was given if one or two of the above correct options were checked and none of the wrong options were checked. A score of 0 was given to any other combination of responses.

#### Independent variables

The independent variables were selected on the basis of substantive and theoretical relevance in accordance with the “*structural influence model of communication in PHEP”*[9]. Our primary independent variable of interest was socioeconomic position (SEP). The following variables were used as primary indicators of SEP: household income, employment status, and educational attainment. Household income was used as a categorical variable (≤$14,999 (reference category), $15,000-$34,999, $35,000-$75,000, and ≥ $75,000).

To further characterize SEP, respondents were asked to report on how often, in the past year, the food they bought ran out and they had no money to buy more. Two categories were created according to the answer given to this question: 1) never (reference category) and 2) sometimes or often. Additional covariates that could influence exposure or attention to information about H1N1 were taken into consideration and included: demographics, neighborhood social cohesion, support of family and friends, having children, living in a specific U.S. region (Midwest, West, Northeast, and South), and language spoken at home. Home ownership was used both as an indicator of SEP and as a social indicator of “participation/investment” in the community and divided into two categories: 1) home owned by self or family and 2) home rented by self or family or being occupied.

Level of educational attainment was considered because of its association with SEP and association to health literacy and knowledge of appropriate health-promoting behaviors [12]. This variable was divided into four categories: 1) less than high school (reference category), 2) high school, 3) some college, and 4) bachelor degree or higher. Employment status was divided into three categories: 1) employed, 2) retired or disabled, and 3) laid off or looking for a job (reference category).

Age was divided into the following categories: 18‐29 (reference category), 30–44, 45–59, and ≥ 60 years. Males served as the reference category for gender. Race/ethnicity was described by the following categories: white, black, Hispanic, and other. Language spoken at home was included because of its impact on media exposure and classified into English and other than English (reference category). U.S. region of residence (Midwest, West, Northeast, and South) was also taken into consideration given potential differences regarding the timing of the start and spread of the pandemic, and the resultant flow of public information.

We measured neighborhood social cohesion using five items asking how strongly respondents agreed that a) “*people around here are willing to help their neighbors,*” b) “*this is a close-knit neighborhood,*”, c) “*people in this neighborhood can be trusted,*” d) “*people in this neighborhood generally don’t get along with each other,*” and e) “*people in this neighborhood do not share the same values.*” We measured responses on a four-point scale and reverse coded the last two items. Even though this measure had demonstrated good internal consistency in previous research [13,14], we retested its reliability in our sample. The results of a principal component analysis identified two factors with eigenvalue > 1, explaining 75% of the total variance. The first factor, describing *community cohesion*, included the first three items reported above accounting for 55% of the total variance; and the second factor, describing *lack of cohesion to the community* included the last two items d) and e), accounting for 20% of the total variance. The first factor showed high internal consistency with Cronbach’s alpha of 0.83, while the second factor showed an alpha value of 0.68. Both factor scores were transformed into a scale ranging from 0 (low cohesion) to 10 (high cohesion) and used as covariates, which were tested both as continuous and categorical (quartiles) variables in our model.

We measured family and friends’ support using three questions addressing the extent to which respondents relied on family and friends to talk or discuss health issues or be advised on health matters. To quantify the extent of such relationships, we used the sum of these three questions.

#### Data analysis

We conducted our statistical analysis using the statistical package STATA version 11.0. We applied survey weights to the data, using the related *svy command* or *svy option* when appropriate, to account for the complex sampling design and to allow estimates to be nationally representative. We also performed descriptive statistical analysis to show the frequency of both the dependent and independent variables. In addition, we applied ordered logistic regression using the *svy ologit* command to test for bivariate associations between each predictor and the two dependent variables: knowledge about H1N1 transmission and knowledge about signs and symptoms of H1N1 (using a p-value ≤0.25 as cut-off)*.* In doing so, we dropped the following variables from the analysis because of p-value > 0.25: gender, U.S. region of residence (Midwest, West, Northeast, and South), family and friends support and “lack of cohesion to the community.*”* We tested the parallel regression assumption by means of the *Brant* test. When the assumption was satisfied, a multivariate ordered logistic model was performed. When not satisfied a generalized regression model was applied. The multivariate procedure included the following indicators of SEP in model 1: household income, level of education, and difficulty in buying food due to the financial situation. In model 2 we added demographics such as age, race/ethnicity, and language spoken at home, while in model 3 we added neighborhood cohesion, house ownership, and parenthood (having children less than 18 years old).

## Results

### Study sample demographics and knowledge levels

We gathered data from the 1,569 subjects completing the survey with a response rate of 66.3%. The weighted sample population included almost equal numbers of men and women, with approximately 50% younger than 44 years of age. Education, income, and language characteristics were: 14% of the population had less than 12 years of education (high school diploma); 14% had an income 100% below the federal poverty level; 22% reported speaking a language other than English at home; and 9% preferred to complete the survey in Spanish. Twenty-nine percent reported being a parent or guardian of one or more children under the age of eighteen. Additional data on the distribution of race/ethnicity, home ownership, neighborhood cohesion, employment status, and financial difficulties among the survey population are shown in 1. Finally, in terms of knowledge of mechanisms of H1N1 virus transmission, forty-four percent checked both correct answers (contact and touching objects), forty-five percent checked at least one of the two correct answers, and eleven percent did not check any correct answer. In terms of knowledge of signs and symptoms of H1N1 infection, sixty-nine percent checked all three correct answers, twenty-four percent checked one or two correct answers, and approximately seven percent did not check any correct answer.

**Table 1:** Distribution of individual socio-demographic characteristics of sample

**Socio-demographic characteristics**	**Weighted estimates of population percentages (%)**
**Gender**	
Female	51.1
Male	49.9
**Age (years)**	
18-29	22.7
30-44	27.2
45-59	28.4
60+	22.7
**Education**	
Less than high school	13.8
High school	29.7
Some college	28.5
Bachelor or higher	27.9
**Parent/guardian of children <18**	
Yes	29.5
No	70.2
**Language spoken at home**	
English	88.7
Other than English	21.3
**Household income ($)**	
≤14,999	13.7
15,000-34,999	20.8
35,000-74,999	34.5
≥75,000	31.0
**Race/ethnicity**	
White	68.3
Black (non-Hispanic)	10.7
Hispanic	14.4
Other	5.2
More than two races (non-Hispanic)	1.2
**Home ownership**	
Owned	71.0
Rented	26.5
Occupied*	2.5
**Employment status**	
Employed	61.0
Retired/disability check	26.1
Laid off/looking for work	12.8
**Food ran out and had no money to buy more**	
Never	76.3
Sometimes	18.9
Often**	4.8
**Neighborhood cohesion**	Mean (SE) = 5.90 (0.10)
	Range = 0-10
**Knowledge about H1N1 virus transmission**	
No correct answer (score = 0)	11
One correct answer (score = 1)	45
Two correct answers (score = 2)	44
**Knowledge about signs and symptoms of H1N1 infection**	
No correct answer (score = 0)	7
One or two correct answers (score = 1)	24
Three correct answers (score = 2)	69

*in the subsequent bivariate and multivariate analysis “*occupied”* was combined with “*rented”.*

**in the subsequent bivariate and multivariate analysis “*often”* was combined with *“sometimes”.*

### Knowledge of H1N1 virus transmission

Table 2 shows the results of the ordered logistic regression analysis performed on the dependent variable *knowledge of H1N1 transmission.* In logistic regression with a single predictor, household income and level of education were individually positively associated with knowledge of H1N1 transmission, while difficulty in buying food due to the financial situation was negatively associated with such outcome. More specifically, for each increase in the category of household income there was an increased likelihood of having a higher level of knowledge. Level of education was also associated with increased knowledge, while those having difficulties in buying food due to their financial situation were less likely to be knowledgeable about H1N1 transmission. Race and ethnicity also were associated with this knowledge, with whites being more likely to have more knowledge than any other group. Blacks and Hispanics in particular were less likely to be knowledgeable about H1N1 transmission than subjects in all other categories. Finally, home ownership was positively associated with greater knowledge, as was living in a home in which English was the only language spoken.

**Table 2:** Ordered logistic regression on knowledge of H1N1 virus transmission

**Independent variables**	**Single predictor models Odds ratios (95% confidence limits)**	**Multiple logistic regression models Odds ratios (95% confidence limits)**		
		**Model 1**	**Model 2**	**Model 3**
Household income	1.24 (1.06-1.45)**	1.08 (0.90-1.31)	1.02(0.84-1.23)	1.01 (0.82-1.24)
Difficulty in buying food due to financial situation	0.53 (0.34-0.81)**	0.64 (0.40-1.03)	0.68(0.42-1.10)	0.70 (0.42-1.17)
Level of education	1.41 (1.20-1.65)***	1.32 (1.11-1.58)**	1.35(1.11-1.61)**	1.35 (1.12-1.63)**
White	2.12 (1.50-3.0)***		2.11(0.82-4.48)	2.11 (0.80-5.54)
Black	0.51 (0.30-0.88)*		0.98 (0.34-2.77)	1.12 (0.38-3.29)
Hispanic	0.55 (0.42-0.72)**		1.69 (0.61-4.68)	1.60 (0.57-4.47)
Language spoken at home rather than English	0.44 (0.32-0.61)**		0.62 (0.33-1.16)	0.81 (0.44-1.48)
Home ownership	1.52 (1.03-2.24)**			0.97 (0.59-1.57)
Parenthood	0.70 (0.48-1.02)			0.73 (0.48-1.11)
Neighborhood cohesion	1.06 (0.98-1.14)			1.03 (0.95-1.11)

*P-value < 0.05.

**P-value < 0.01.

***P-value < 0.001.

Parenthood and neighborhood cohesion did not have a statistically significant association with knowledge levels about H1N1 transmission. Of the multiple logistic ordinal regression models with more than one predictor, model 1 indicated that only level of education remained a significant predictor of this knowledge. This finding suggested that the other two SEP variables identified previously as significant predictors were correlated with education. In model 2, which controlled for several demographic characteristics including race/ethnicity and language spoken at home, level of education still remained a strong predictor of knowledge about virus transmission. Similar results were obtained by introducing home ownership, parenthood status, and neighborhood cohesion into model 3.

### Knowledge about signs and symptoms of H1N1 infection

In Table 3, the results of the logistic regression models are presented for “*knowledge about signs and symptoms of H1N1 infection”* as the dependent variable*.* In the single predictor models, race/ethnicity, age, language spoken at home, home ownership, and community cohesion (trust in the community) all individually demonstrated a statistically significant association with this variable. As shown here, whites showed greater likelihood of being at a higher level of knowledge than non-whites. Conversely, Hispanics showed decreased likelihood as did respondents speaking a language other than English at home. Those who own a home were most likely to have higher levels of knowledge about signs and symptoms of infection, and for each unit increase in community cohesion (0–10 scale) we found an increased likelihood of being at a higher level of knowledge as well.

**Table 3:** Generalized ordered logistic regression on knowledge about signs and symptoms of H1N1 infection

**Independent variables**^**#**^	**Bivariate**	**Model 1**	**Model 2**	**Model 3**
	**Odds ratios (95% C.I.)**	**Odds ratios (95% C.I.)**	**Odds ratios (95% C.I.)**	**Odds ratios (95% C.I.)**
Household income	1.17 (0.97-1.42)	1.10 (0.87-1.40)	1.10 (0.86-1.41)	0.98 (0.75-1.30)
Difficulty in buying food due to financial situation	0.65 (0.42-1.01)	0.71 (0.43-1.17)	0.83 (0.49-1.39)	0.89 (0.52-1.53)
Level of education	1.11 (0.93-1.34)	1.04 (0.83-1.30)	1.03 (0.81-1.31)	1.04 (0.82-1.32)
Age	1.29 (1.07-1.55)**		1.24 (1.02-1.52)*	1.21 (0.98-1.49)
White	1.57 (1.08-2.28)*		1.27 (0.71-2.26)	1.15 (0.65-2.03)
Hispanic	0.62 (0.46-0.83)**		1.17 (0.58-2.39)	1.13 (0.54-2.34)
Language spoken at home rather than English	0.53 (0.38-0.74)***		0.66 (0.35-1.26)	0.78 (0.40-1.50)
Home ownership	1.90 (1.26-2.86)**			2.89 (1.26-6.66)*^1^
				1.40 (0.82-2.38)^2^
Neighborhood cohesion	1.09 (1.05-1.14)***			1.10 (0.99-1.20)

#: black and parenthood were not reported in this table because of p-value >0.25.

1: knowledge score 2 versus 0.

2: knowledge score 2 versus 1.

*P-value < 0.05.

**P-value < 0.01.

***P-value < 0.001.

Age was also associated with knowledge about signs and symptoms, with increased likelihood of higher knowledge per increased category of age. In the regression models with multiple predictors, the Brant test for the parallel regression assumption showed a violation of the assumption for two variables: difficulty in buying food due to the financial situation (Brant test p-value = 0.03) and neighborhood cohesion (Brant test p-value = 0.01). We therefore applied a generalized ordered logistic regression model in the multivariate procedure using the *gologit2 (svy autofit option)* command in STATA. Differences by category of knowledge appeared only in the final model (model 3). In model 1, similar to the analysis performed on knowledge about H1N1 virus transmission, we included the following SEP indicators: household income, difficulty in buying food due to the financial situation, and level of educational attainment. None of these variables had a statistically significant association with the outcome. In model 2, we added demographic variables including race/ethnicity, age and language spoken at home (other than English). In this case, only age was significantly associated with increased likelihood of being at a higher level of knowledge with all other variables held constant. In model 3, we added home ownership and neighborhood cohesion to the model—the only case where we found differences by category of knowledge that justified using the generalized model. When the highest level of knowledge (knowledge score = 2) was compared to the lowest one (knowledge score = 0), respondents who owned a home showed increased likelihood of being at a higher level of knowledge than those who did not own a home. This result was statistically significant (p-value = 0.01). On the contrary, the association was not confirmed to be significant (p-value = 0.21) when the highest category of knowledge (knowledge score =2) was compared to the middle one (knowledge score = 1).

### Study strengths and limitations

The response rate for this survey was 66.3% (51% for the Hispanic population), a good rate for RDD surveys. For this reason we can assume that in our study, the response bias, defined as the difference between the observed value from a survey, and the value that would have been observed given no response, is acceptable and that results can be generalized to the source population [15]. Hence, inference to the U.S. population can be made on the association we found between knowledge of H1N1 and SEP, level of education, and home ownership.

Because we used a cross sectional study design, however, the timing of the survey must be considered in interpreting and generalizing the results. The study was fielded in March 2010 when most of the population had been exposed to some sort of information about H1N1 for months. Also, most of the subjects surveyed had already made decisions on the need to follow preventive measures [16]. There may therefore have been even greater gaps in knowledge across social groups at an earlier time in the epidemic when there was more uncertainty on the development of the outbreak and more limited exposure to information.

In terms of statistical analysis, our chosen technique also has some limitations. Most notably, while ordered logistic regression is technically appropriate for an ordered outcome of this sort, it’s important to remember that the ORs are not percentages but, rather, a ratio of two different odds (each representing the *odds* of an event occurring, i.e., is the probability of the event divided by the probability of an event *not* occurring), and describe the strength of the association between two binary data values in a symmetric way. If misinterpreted as percentages, however, the ORs could overestimate risks given the frequencies of the outcomes [17,18].

## Discussion

Public health messages are often subject to differences in interpretation that can vary considerably according to individual perception of the risk and trust in the government as well as according to different abilities to understand and interpret data and information, especially in the context of uncertainty [19,20]. During the H1N1 pandemic, federal, state, and local public health agencies faced these kinds of challenges in their efforts to provide clear information and advice to the public while at the same time balancing what was known and not known on the outbreak. Public health officials faced with the task of disseminating infection control messages to the public in the context of sustained media coverage often had limited knowledge about how the information would reach the population and the population’s ability to learn and act upon it. Under these circumstances, public officials in both the U.S. and abroad had no choice but admit the lack of science supporting policy recommendations and modify such recommendations once more evidence became available [21].

The results of our study show how SEP in general, and level of education in particular, of the intended recipients of this public health information were strongly associated with their level of knowledge about H1N1. These results in turn suggest that increased overall level of education can prepare people for receiving and processing more complex information, even under conditions in which elements of uncertainty or changes over time complicate the message and the nuances of interpreting it. Such findings are consistent with the structural influence model developed by Viswanath et al. (2009) [9] that posits a relationship between SEP, communication, and health outcomes, with formal education standing out as a strong predictor influencing still-evolving knowledge about a complex topic such as a pandemic. The association between level of education and knowledge about H1N1 found in this study, in fact, suggests that even after ten months into the pandemic, messages about virus transmission did not reach the less educated. The data also suggest that public health officials should take into account differences in population subgroups as they develop public communication strategies, lest they exacerbate the inequalities that already exist among the groups. Public officials need to develop methods and strategies (i.e., rapid surveys) to test their messages and assess their impact on the population.

This study also showed that another SEP factor, home ownership, was related to knowledge about H1N1. In previous studies, home ownership has been shown to be an important predictor of good health. Those who live in rented living spaces have more symptoms and long-term illness, and they report poorer general and mental health than owner-occupiers [22-25]. In times when over 2.5 million homes received a foreclosure filing, finding an association between knowledge of H1N1 and home ownership raises concerns and brings attention to the importance of taking into consideration yet another economic indicator in communication planning efforts. Furthermore, home ownership is not only an indicator of economic status but of social cohesion as well. Owning a home anchors individuals to the community in which they live, and neighbors serve as one potential source of information [26] and knowledge. Moreover, better integration into the community is likely to be related to greater trust in local authorities, which in turn may affect willingness to heed the message and consequent ability to learn from information received. Last, home ownership provides access to resources in the neighborhood. This suggests that in areas where house-ownership is limited, public health officials may want to design more creative public communication strategies. These might include developing messages that rebuild that feeling of trust in the government, as well as selecting non-governmental channels of communication, such as community-based organizations, community leaders, or family networks, to ensure the diffusion of their messages among all social groups. Also, given the significant role of formal schooling, channels such as local television that are watched by people with less formal education could be an important platform to deliver messages. Further research that aims to elucidate how different segments of the population access information in times of crisis and learn and act upon it is needed to provide public health officials with a better understanding of the population they serve, help them to capitalize on existing cultural differences, and ultimately shape better communication capabilities for complex emergency situations.

## Conclusions

Our study results show that during the H1N1 pandemic communication inequalities in the U.S. population correlated with differences in knowledge of H1N1 in terms of virus transmission and signs and symptoms of infection. The relationship between this knowledge and both level of education and home ownership found in this study suggests the need for public officials to integrate information about the characteristics of communities and individuals, with a particular focus on SEP in their communication planning efforts.

## Competing interests

The authors declare they have no competing interests.

## Pre-publication history

The pre-publication history for this paper can be accessed here:

http://www.biomedcentral.com/1471-2458/12/328/prepub
